# Correlation between dipstick urinalysis and urine sediment microscopy in detecting haematuria among children with sickle cell anaemia in steady state in Ilorin, Nigeria

**DOI:** 10.11604/pamj.2013.15.135.1854

**Published:** 2013-08-15

**Authors:** Emmanuel Ademola Anigilaje, Olanrewaju Timothy Adedoyin

**Affiliations:** 1 Department of Paediatrics, Federal Medical Centre, Makurdi, Benue State, Nigeria; 2 University of Ilorin Teaching Hospital, Ilorin, Nigeria

**Keywords:** Sickle cell nephropathy, children, haematuria, dipstick urinalysis, urine sediment microscopy

## Abstract

**Introduction:**

Haematuria is one of the clinical manifestations of sickle cell nephropathy. Although dipstick urinalysis detects haemoglobin and by extension haematuria; it does not confirm haematuria. Urine sediment microscopy confirms haematuria and constitutes a non-invasive “renal biopsy”. The need to correlate dipstick urinalysis and urine sediment microscopy findings becomes important because of the cheapness, quickness and simplicity of the former procedure.

**Methods:**

Dipstick urinalysis and urine sediment microscopy were carried (both on first contact and a month after) among consecutive steady state sickle cell anaemia children attending sickle cell clinic at the University of Ilorin Teaching Hospital between October 2004 and July 2005.

**Results:**

A total of 75 sickle cell anemia children aged between 1-17 years met the inclusion criteria. Haematuria was found in 12 children (16.0%) and persistent haematuria in 10 children 13.3%. Age and gender did not have significant relationship with haematuria both at first contact (p values 0.087 and 0.654 respectively) and at follow-up (p values 0.075 and 0.630 respectively). Eumorphic haematuria was confirmed in all the children with persistent haematuria with Pearson correlation +0.623 and significant p value of 0.000.

**Conclusion:**

The study has revealed a direct significant correlation for haematuria detected on dipstick urinalysis and at urine sediment microscopy. It may therefore be inferred that dipstick urinalysis is an easy and readily available tool for the screening of haematuria among children with sickle cell anaemia and should therefore be done routinely at the sickle cell clinics.

## Introduction

Sickle cell nephropathy is an important cause of morbidity and mortality in patients with sickle cell anaemia [1]. It manifests clinically as proteinuria with or without nephrotic syndrome, immune-complex glomerulonephritis, progressive renal failure, impaired urinary concentration ability, incomplete distal tubular acidosis and haematuria [2]. Haematuria may occur continually as a low grade haematuria and or in episodic gross haematuria [3] and may occur at any age [4] Although dipstick urinalysis remains a valuable tool in the screening of haematuria, it does not confirm haematuria.[5] It uses a peroxidase-like chemical reaction between hemoglobin or myoglobin and a chemical indicator compound [5] It therefore cannot differentiate clearly between myoglobin and haemoglobin. More also, haemoglobinuria can occur without haematuria as may occur in haemolytic anaemia [5]. Urine sediment microscopy is useful in this regard. By detecting erythrocytes, it confirms a positive dipstick reaction as haematuria. Urine sediment microscopy also differentiates the origin of haematuria within the urinary tract system [5, 6]. For example, upper urinary tract source originating from the glomerulus reveals red cell casts and 75% or greater of the urinary erythrocytes are deformed (dysmorphic erythrocytes) [5, 6] Haematuria originating within the convoluted/collecting tubules may be associated with leucocyturia or renal tubular cell casts [5]. On the other hand, lower urinary tract haematuria (pelvocalyceal system, ureter, bladder or urethra and interstitium) is associated with eumorphic erythrocytes with normal morphology and fewer 25% or less may be dysmorphic [5, 6]. Urine sediment microscopy therefore constitutes a non-invasive assessment of the renal parenchymal morphology.[7] If significant correlation does exists between dipstick urinalysis and urine sediment microscopy in the detection of haematuria, dipstick urinalysis may then suffice in identifying patients with haematuria, who may further benefit from further renal functions tests including glomerular filtration rate assessment.

## Methods

A prospective study in which consecutive sickle cell anaemia patients (HbSS) who came for routine follow-up clinic were recruited. Two clinical contacts were required, the second contact being a month after the first one. Clearance was obtained from the Hospital’s Ethics and Research Committee. Informed consent was obtained from all subjects and or parents/legal care providers as appropriate. The inclusion criteria included children between nine months and eighteen years with haemoglobin SS as confirmed by electrophoresis using the cellulose acetate paper. They were assumed to be in the steady state when they satisfy the following criteria; [8] (i) No fever at presentation and for 4 weeks preceding clinical attendance,(ii) No complaints of skeletal and or abdominal pain at presentation and within the 4 weeks preceding the investigation (iii) Not on any medication apart from routine folic acid and proguanil (iv) Otherwise well and going about their routine activities. Exclusion criteria included subjects with (i)Symptoms and signs suggestive of urinary tract infections (ii)A history of exposure to radio-opaque dye and some drugs that decrease the reactivity of dipstick reagents including nitrofurantoin, cephalexin, cephalothin, captopril, tetracycline - 4 weeks earlier [9], (iii) menstruation or vaginal/penile discharge [9], (iv) Fever [5], (v) Involvement in competitive sport/exercise in the previous 24 hours [5].

All enrolled subjects were provided with a properly labeled universal bottle for the collection of early morning urine. Subjects were instructed on how to collect early morning mid-stream urine depending on the age. The care givers collected for children less than 4 years. The investigator was available as early as 7.00 a.m. to receive urine specimens. Early morning urine is expected to be concentrated and therefore most suitable for microscopy and biochemical analyses. When tests could not be performed within the first hour of urine collection, urine was stored in the refrigerator (at 4°c) and tested within two hours of storage in the refrigerator. Urine refrigerated was kept at room temperature for 15 minutes before tests were performed.

Dipstick urinalysis was done using Multistix 10SG by Bayer Diagnostic [9]. All instructions regarding the storage and handling of reagent strip were observed as stipulated by the manufacturer. The various grades of findings on colour chart (and their significant quantitative values) were recorded as follows; negative, trace (10 erythrocytes/µl), small +( 25 erythrocytes/ µl), moderate 2+ (80 erythrocytes/ µl) and large 3+ (200 erythrocytes / µl).(9)

Urine sediment microscopy procedure was carried out as thus [10]; a sample of well mixed urine (usually 10 mls was centrifuged in a test tube at a relatively low speed (3000rpm) for 5 minutes. The supernatant was decanted and a volume of 0.2 to 0.5ml was left inside the tube. The sediment was re-suspended in the remaining supernatant by flicking the bottom of the tube several times. A drop of re-suspended sediment was put on a glass slide and a cover slip applied. The slide was first examined under the lower power objective of the microscope for identification of most casts. Next examination was carried out at high power objective to identify erythrocytes.10 to 15 fields were examined under each low and high power objective. Casts were recorded as average number of casts seen per low power field. Red blood cells were recorded as average of red blood cells per high power field. To enhance proper identification of the shape and sizes of urinary red cells, a Wright staining of the air dried smear of urinary sediments was carried also carried out.

To ensure quality control, the investigator had been tutored on the procedures of dipstick urinalysis and urine sediment microscopy for 3 months (tutored by a consultant microbiologist and a senior laboratory technologist), including practical demonstrations at the microbiological laboratory of the University of Ilorin Teaching Hospital before the commencement of the study. Slides of findings of the urine sediment microscopy were also verified by the tutors.

Subjects were grouped into increasing age-groups of four (1 -5, 6-10, 11-15, and ≥ 16 years). The prevalence of haematuria was calculated on the first contact and at follow-up. Frequency table with percentages were generated for haematuria for different age groups and gender. Chi square (x^2^) test was adopted to test for association between age groups and gender and haematuria. Pearson's correlation coefficient was adopted to test the correlation between haematuria on dipstick urinalyses and urine sediment microscopy on the first contact and at follow-up. The quantitative equivalents of haematuria on dipstick urinalysis were adopted before Pearson's correlation coefficient was employed. Epi-info Software package (version 6.04) and SPSS 11.1 were for data analysis. P value of < 0.05 was regarded as significant.

### Definition of terms

**Dipstick urinalysis:** Haematuria; presence of trace or more blood in the urine. Significant haematuria; presence of (1+) or more blood in the urine. Persistent haematuria; a situation whereby haematuria was found on first contact and at follow-up (1 month after the first contact)

**Urine sediment microscopy:** Haematuria; presence of 2 or more red blood cells. Leucocyturia; presence of white blood cells. Dysmorphic haematuria; presence of irregular, non-uniform sized red blood cells with in-homogenous cytoplasm in the urine. Eumorphic haematuria; the presence of regular, uniform sized red blood cells with homogenous cytoplasm. Casturia; presence of a cast per low power field.

## Results

A total of 80 sickle cell anaemia children met the inclusion criteria but 75 were evaluated at follow-up. Five subjects were lost to follow-up. The 75 children comprised 35 males and 40 females with a male to female ratio of 1:1.1. The age range was between 1-17 years with a mean age of 8.9± 4.5 years. The age group and gender distribution of the subjects are as shown in Table 1. Prevalence rate for haematuria on first contact was 16.0% and 13.3% at follow-up, while that of significant haematuria on first contact and at follow-up was 2.6%. The prevalence rate for persistent haematuria was 13.3%. Gender was not found to have significant relationship with haematuria on first contact (x^2^=0.848, df=2 p value=0.654) and at follow-up ( x^2^=0.93, df=2, p value=0.630). Age also did not have significant relationship with haematuria on first contact (x^2^=11.06, df=6, p value=0.087) and at follow-up (x^2^=14.49, df=6, p value=0.075) Table 2. There was no urinary abnormality consistent with nephritic, nephrotic and renal failure. The urine of the eleven year old girl (S/N 19) who had significant leucocyturia also yielded significant growth of *Escherichia coli* that was sensitive to co-trimoxazole, cefuroxime, gentamycin and nitrofurantoin ( prevalence of 1.3% for asymptomatic bacteriuria) Table 3. There was a significant positive correlation between haematuria found on dipstick urinalyses and that of urine sediment microscopy Table 4.

**Table 1:** Age group and gender distribution of subjects

Age group (years)	Male	Female	Total	%
<5	10	12	22	29.3
6-10	11	15	26	34.7
11-15	10	8	18	24
≥16	4	5	9	12
Total	35	40	75	100

**Table 2:** Haematuria on Dipstick Urinalysis for Subjects on First Contact and at Follow-up

Age group (years)	No Haematu		Haematu		Significant Haematu		Persistent Haematu	
	M	F	M	F	M	F	M	F
1-5	9(10)	11(11)	1(0)	1(0)	0(0)	0(0)	0	1
6-10	10(10)	14(15)	1(1)	1(0)	0(0)	0(0)	1	0
11-15	7(7)	6(6)	3(3)	2(2)	1(1)	1(1)	3	2
≥ 16	2(2)	4(4)	2(2)	1(2)	0(0)	1(1)	2	1
Total	28(29)	35(36)	7(6)	5(4)	1(1)	1(1)	6	4
Prevalence rate %	37.3(38.7)	46.7(48)	9.3(8)	6.7(5.3)	1.3(1.3)	1.3(1.3)	8	5

M= Male, F= Female; No Haematu=No haematuria found; Haematu=haematuria found; Significant Haematu= Significant haematuria found; Persistent Haematu= Persistent haematuria found; Figures in parenthesis were findings at follow-up; Please note that subjects with significant Haematu and those with Persistent Haemtu were part of those with Haematuria; Relationship between gender and haematuria on first contact (x^2^=0.848, df=2 p value=0.654) and at follow-up; (x^2^=0.93, df=2, p value=0.630). Relationship between age and haematuria on first contact (x^2^=11.06, df=6, p value=0.087) and at follow-up; (x^2^=14.49, df=6, p value=0.075).

**Table 3:** Urine Sediment Microscopy for Fifteen Subjects with Urinary Abnormalities

S/N	Age(years)	Gender	RBC/HPF	WBC/HPF
4	5	F	3(4)	Nil (Nil)
8	5	M	4(Nil)	Nil (Nil)
9	10	M	3(4)	Nil (Nil)
12	10	F	5(Nil)	Nil (Nil)
16	11	M	3(5)	5(7)
19	11	F	Nil (Nil)	6(5)
21	11	F	2(2)	Nil (Nil)
24	12	M	Nil (Nil)	Nil (Nil)
30	13	F	3(4)	Nil (Nil)
35	13	M	2(4)	5(4)
40	14	M	4(5)	Nil (Nil)
46	16	F	Nil (Nil)	5(6)
55	16	M	4(4)	Nil (Nil)
60	17	M	6(4)	Nil (Nil)
65	17	F	5(6)	1(2)

S/N=serial number of subjects, M=Male, F=Female, HPF=High power field, RBC=Red Blood Cells, WBC=White Blood Cells; Figures in parentheses are findings at follow-up; Additional findings; Amorphous phosphate crystals were found in subject S/N 16 on first contact. Granular casts were found in subject S/N 16 on first contact and at follow-up. A few yeast cells were also found in subject S/N 16 on first contact and at follow-up

**Table 4:** Correlation between dipstick urinalyses and microscopy at first contacts and at follow-up

	Pearson correlation (r)	p-value
Haematuria	+0.620	0.000
Haematuria	+0.623	0.000

Word/figures in italics represent findings at follow-up

## Discussion

The prevalence of haematuria of 13.3% among sickle cell anaemia children differed from those of Ocheke [11] and Aikhionbare et al [12] who did not detect haematuria in any of the 22 and 101 sickle cell anaemia patients respectively. Furthermore, the prevalence of 2.1% reported by Konotey-Ahulu [13] among 1,347 sickle cell anaemia patients was lower than the prevalence of 13.3% found in this study. However, the prevalence of persistent haematuria of 13.3% in this study compared to those of Ugwu and Eke [14] who found a prevalence of 11% among 72 sickle cell anaemic children. Whereas the small sample size [22] in the study of Ocheke [11] may be responsible for the absence of haematuria among the sickle cell anaemia children, the reason for the disparity in prevalence of haematuria between this study and that of Aikhionbare et al [12] is not easily discernible. However, the hitherto recognized observers’ differences in the reading of dipstick urinalysis cannot be ignored.

Although there was an apparent increase in the prevalence of haematuria with increasing age (for those over the age of 10 years), this was not significant. Ugwu and Eke [14] and Konotey-Ahulu [13] also reported increased prevalence of haematuria in children older than 5 years, even though they did not set out to study the relationship between age and haematuria. Furthermore, haematuria occurred more among male than female subjects, both on the first contact and at follow-up, these observations were not significant and probably reflect the fact that more male than female subjects were seen during the study period. The absence of significant relationship between gender and haematuria is unexpected as the patho-physiology of sickle cell nephropathy is similar regardless of gender. Repeated gross or microscopic haematuria has sometimes been detected among sickle cell anaemia children, usually involving the left kidney and at times severe enough to endanger the patient's life or be confused with renal malignancy.[4] In this study, the percentage of patients with persistent haematuria on dipstick urinalysis and urine sediment microscopy was relatively high (13.3%). The microscopic haematuria was basically of non-glomerular origin as the red blood cells were of uniform sizes, shapes and of homogenous cytoplasm. This eumorphic haematuria may result from renal papillary necrosis. It has been proposed that that increased sickling of erythrocytes in the renal medulla results in necrosis of the renal papilla and subsequent extravasations of blood into the urine [5]. The acidic environment, its low PaO_2_ (35-40mmHg) lying below sickling threshold (40mmHg) as well as high osmolality are factors known to promote sickling in the medulla [4]. Dysmorphic haematuria can also occur from membrano-proliferative glomerulonephritis and glomerular membrane splitting [4].

## Conclusion

The study has revealed a direct significant correlation for haematuria detected on dipstick urinalysis and at urine sediment microscopy. It may therefore be inferred that dipstick urinalysis is an easy and readily available tool for the screening of haematuria among children with sickle cell anaemia and should therefore be done routinely at the sickle cell clinics. If haematuria and other urinary abnormalities are detected with dipstick urinalysis, further renal functions tests can be done to identify such children with early parenchymal disease and efforts aimed at retarding progression of renal impairment i.e, control of hypertension, moderate protein intake, and the use of angiotension-1- converting enzyme inhibitors [15] may then be instituted.
